# PAGER-scFGA: unveiling cell functions and molecular mechanisms in cell trajectories through single-cell functional genomics analysis

**DOI:** 10.3389/fbinf.2024.1336135

**Published:** 2024-04-16

**Authors:** Fengyuan Huang, Robert S. Welner, Jake Y. Chen, Zongliang Yue

**Affiliations:** ^1^ Department of Biomedical Informatics and Data Science, School of Medicine, University of Alabama at Birmingham, Birmingham, AL, United States; ^2^ Hematology & Oncology, School of Medicine, University of Alabama at Birmingham, Birmingham, AL, United States; ^3^ Health Outcome Research and Policy Department, Harrison College of Pharmacy, Auburn University, Auburn, AL, United States

**Keywords:** PAGER, functional genomics analysis, network biology, single-cell analysis, natural killer cell and GNPA

## Abstract

**Background:** Understanding how cells and tissues respond to stress factors and perturbations during disease processes is crucial for developing effective prevention, diagnosis, and treatment strategies. Single-cell RNA sequencing (scRNA-seq) enables high-resolution identification of cells and exploration of cell heterogeneity, shedding light on cell differentiation/maturation and functional differences. Recent advancements in multimodal sequencing technologies have focused on improving access to cell-specific subgroups for functional genomics analysis. To facilitate the functional annotation of cell groups and characterization of molecular mechanisms underlying cell trajectories, we introduce the Pathways, Annotated Gene Lists, and Gene Signatures Electronic Repository for Single-Cell Functional Genomics Analysis (PAGER-scFGA).

**Results:** We have developed PAGER-scFGA, which integrates cell functional annotations and gene-set enrichment analysis into popular single-cell analysis pipelines such as Scanpy. Using differentially expressed genes (DEGs) from pairwise cell clusters, PAGER-scFGA infers cell functions through the enrichment of potential cell-marker genesets. Moreover, PAGER-scFGA provides pathways, annotated gene lists, and gene signatures (PAGs) enriched in specific cell subsets with tissue compositions and continuous transitions along cell trajectories. Additionally, PAGER-scFGA enables the construction of a gene subcellular map based on DEGs and allows examination of the gene functional compartments (GFCs) underlying cell maturation/differentiation. In a real-world case study of mouse natural killer (mNK) cells, PAGER-scFGA revealed two major stages of natural killer (NK) cells and three trajectories from the precursor stage to NK T-like mature stage within blood, spleen, and bone marrow tissues. As the trajectories progress to later stages, the DEGs exhibit greater divergence and variability. However, the DEGs in different trajectories still interact within a network during NK cell maturation. Notably, PAGER-scFGA unveiled cell cytotoxicity, exocytosis, and the response to interleukin (IL) signaling pathways and associated network models during the progression from precursor NK cells to mature NK cells.

**Conclusion:** PAGER-scFGA enables in-depth exploration of functional insights and presents a comprehensive knowledge map of gene networks and GFCs, which can be utilized for future studies and hypothesis generation. It is expected to become an indispensable tool for inferring cell functions and detecting molecular mechanisms within cell trajectories in single-cell studies. The web app (accessible at https://au-singlecell.streamlit.app/) is publicly available.

## Introduction

Single-cell analysis methods provide unprecedented opportunities to unravel the cell, providing a high-resolution identification of cells. This is crucial for investigating molecular events in complex diseases, such as those affecting the immune system (Griffiths et al., 2018; Ginhoux et al., 2022; Xu et al., 2022), neurological disorders (Mathys et al., 2019; Arenas, 2022), and cancers (Patel et al., 2014; Le et al., 2020). The conventional single-cell sequence analysis framework followed by the four major procedures, tissue dissociation, single-cell RNA sequencing, bioinformatics analysis and experimental validation to yield insights into the associations between the genotype and phenotype (Chavkin and Hirschi, 2020). However, the cell type annotation and functional genomics analysis are *ad hoc* procedures in this analysis framework. Moreover, the lack of in-depth functional genomics technology with systems biology impedes the potential biological interpretation of the results.

Functional genomics analysis serves as a pivotal tool for unraveling the intricate interplay of genes, proteins, and small molecules, orchestrating specific functions in response to both extrinsic and intrinsic factors, such as small molecules or secreted proteins. Functional genomics has witnessed notable success in advancing disease diagnosis (Zhang and Chen, 2010; Drier et al., 2013; Livshits et al., 2015; Bock and Ortea, 2020; Pian et al., 2021), cancer subtyping (Zhang and Chen, 2013; Mallavarapu et al., 2020; Lafferty et al., 2021), and personalized medicine (Chen et al., 2007; Hamburg and Collins, 2010; Raghavan et al., 2017). To gain a profound understanding of the molecular mechanisms underpinning diseases or contributing to various disorders, comprehensive functional genome analysis has emerged, encompassing genomics, epigenomics, proteomics, and interactomics. In order to describe gene and protein functions under specific biological conditions or treatments, the integration of geneset, network, and pathway analysis (GNPA) has proven instrumental (Yue et al., 2015; Yue et al., 2018; Yue et al., 2022). Pathway analysis, particularly topology-based approaches leveraging the knowledge about gene and protein interactions within pathways, has been developed to unveil mechanistic changes through pathway-level scoring and pathway significance assessment. To aid in functional inference for new single-cell datasets, the CellMarker database (Zhang et al., 2019; Hu et al., 2023) has been introduced, providing valuable cell-type-specific gene signatures. In conjunction with single-cell analysis methods, the field of functional genomics opens up an exciting realm of opportunities for exploring cellular-level mechanisms and expanding our knowledge.

In this work, we develop PAGER-scFGA, a comprehensive tool featuring cell-type functional inference and network-based analysis through cell trajectories. PAGER-scFGA facilitates the functional annotation of cell groups, unravels the functional changes within the pathways and pathway-pathway cross-talk network in each trajectory, and characterizes up/downregulated gene functional compartments (GFC) underlying cell maturation and differentiation. In our mNK cell study, we enhance the current understanding of NK cells from the cell subtype and the cell stage in maturation/differentiation. To facilitate the usage of our data for the wide research community, an interactive portal has been developed to analyze and visualize our mNK cells. We envision PAGER-scFGA to be a powerful tool in the study of single-cell functional genomics analysis in complex diseases.

## Materials and methods

### Single-cell collection and sequencing for mouse samples

Single cells were obtained from mouse bone marrow, blood, and spleen tissues. The isolation of mouse natural killer (mNK) cells was carried out using the CITE-seq (Cellular Indexing of Transcriptomes and Epitopes by Sequencing) method, which involved the utilization of antibodies targeting CD11b, CD27, NK 1.1, TCRb, and CD122. This process was performed using three 10x Genomics single-cell lines, each incorporating three distinct hashtags (antibodies) to differentiate the tissue sources.

### Single-cell RNA-seq data processing

The mouse single-cell samples were processed using Cell Ranger v4.0.0, and read alignment was performed with the GRCm38 mouse genome. We conducted filtering to remove low-quality cells and genes based on two criteria: (1) cells with expressed gene (counts larger than 0) less than 200, and (2) expressed genes less than three cells. Since all the samples were processed using the same 10x Genomics platform, there was no need for batch effect correction.

### PAGER-scFGA framework

The PAGER-scFGA framework has been harnessed to create a publicly accessible web application tailored for the analysis of mouse natural killer cells, known as the PAGER-scFGA app. The app’s Git repository is found at https://github.com/zongyue1010/au-singlecell. This interactive platform is composed of five major sections, seamlessly integrating the Scanpy library, the Streamlit library, and the PAGER functional genomics analysis API.

**FIGURE 1:**
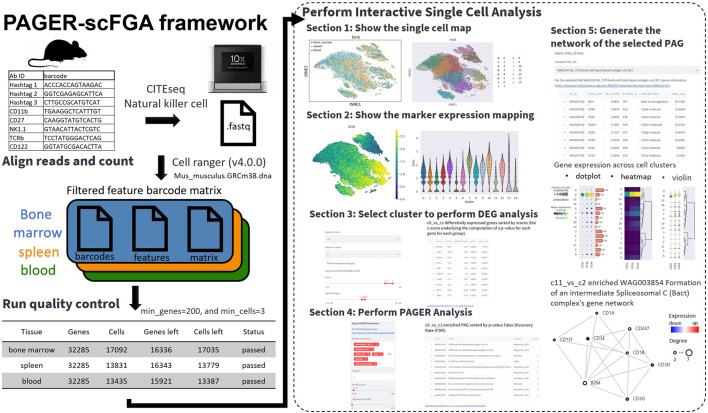
The overview of the PAGER-scFGA framework. The preprocessing of the single cells with the CITE-seq with the antibody-tagged barcodes (left) and the interactive single-cell analysis using the PAGER-scFGA (right). We applied the CITE-seq data from mouse natural killer cells. We performed a cell ranger to generate the feature barcode matrix and run quality control using the standard cutoffs, with the minimum genes set to 200 and the minimum cells set to 3. The interactive single-cell analysis consists of five major sections.

We employed single-cell data from mNK cell samples to showcase the capabilities of the scFGA app (Figure 1). The application is designed as follows:

1. Visualization of Single-Cell Maps: The first section allows users to view single-cell maps through t-SNE or UMAP plots, with tissue types color-coded and cluster annotations numbered. The tool enables users to refine their exploration by filtering clusters; they can deselect specific cluster numbers to regenerate plots. This action triggers dynamic updates to both the selected and reference cluster/clusters. Moreover, users can delve deeper into the analysis of differentially expressed genes (DEG) in Section 3 by selecting specific clusters and reference cluster/clusters.
2. Marker Expression Visualization: In the second section, users can explore marker expression in the t-SNE/UMAP plot and assess the expression differences of selected genes across various clusters.
3. DEG Extraction: The third section empowers users to select a cluster and a reference cluster (or all remaining clusters) to extract DEGs using Wilcoxon rank-sum (Mann-Whitney-U) test as recommended by the paper (Soneson and Robinson, 2018) in default. The scFGA app considers user-defined cutoffs for scores underlying the computation of the *p*-value for each gene for each group, log fold changes, and adjusted *p*-value, generating a table of genes meeting these criteria.
4. PAGER Analysis: In the fourth section, the scFGA app performs PAGER analysis and presents all enriched PAGs based on user-defined parameters, including data source, overlap, similarity score, and -log2-based FDR cutoff. Users can further refine the results by adjusting the PAG size range. Resulting P values were adjusted for multiple testing for each factor using the Benjamini–Hochberg procedure (Benjamini and Hochberg, 2018). The tables depicting m-type and r-type PAG-to-PAG relationships among the filtered PAGs in the PAGER database are generated or updated accordingly.
5. Gene Network Generation: In the fifth section, users can select a specific PAG ID to generate a gene network. This section provides three downloadable tables, including detailed information about PAG gene members, DEG results from section three alongside mapped mouse genes to human homologs (utilizing 1-to-1 best homologs from NCBI Homologene build 68), and the genes shared between DEGs and PAG gene members. The scFGA app offers an interactive network featuring weighted genes based on network topology (RP-score) (Yue et al., 2019; Yue et al., 2021), with colored nodes representing expression levels. The gene network was constructed by extracting Protein-Protein Interactions from the HAPPI two database (Chen et al., 2017), with a stringent quality threshold of no less than three stars (confidence score ≥0.45). Additionally, we incorporated the generation of the network using Protein-Protein Interactions from the STRING database (Szklarczyk et al., 2023), employing a stringent quality threshold of no less than 0.7.

Links for downloading tables are conveniently provided below each of them.

### The cell type functional inference based on the enrichment analysis of DEGs in the cell clusters

To perform cell type inference through enrichment analysis, we incorporated cell type gene signatures from the CellMarker database (Zhang et al., 2019; Hu et al., 2023), which provided us with access to 534 distinctive gene signature sets. Leveraging these sets, we applied enrichment analysis to the cell cluster-specific DEGs, identifying the most significantly enriched cell type gene signature set. This set was then utilized for functional inference, facilitated through the PAGER API, with the data source specified as “CellMarker".

### The construction of protein-protein interaction (PPI) network from DEGs and the gene subcellular map

After identifying the DEGs in the selected cluster and comparing them to the reference clusters within the trajectory (detailed in the **Supplemental**), we proceeded to retrieve protein-protein interactions from the STRING database (Szklarczyk et al., 2023), employing a confidence score cutoff of 0.40, which represents a moderate confidence level. Subsequently, we obtained information on gene cellular components from the Gene Ontology database. By considering the Gene Ontology annotations of the selected genes and their neighboring genes within the constructed PPI network, we inferred the subcellular locations of these genes. This classification encompassed extracellular space, membrane, cytosol, and nucleus, as documented in Supplementary Table S1. Furthermore, based on the gene functions as reported in GeneCard (Stelzer et al., 2016) and WikiGene (Hoffmann, 2008), we categorized genes with similar functions into distinct gene functional compartments (GFCs).

### Pseudotime inference and the signal curve plot of GFCs in trajectory

The differentiation trajectory of all mNK cells was inferred using the diffusion map algorithm following the Scanpy workflow (Haghverdi et al., 2016). We chose the c2 NK cells as the root due to their higher proportion among bone marrow mNK cells. The diffusion pseudotime was calculated using the scanpy. tl.dpt function. To generate the signal curve along pseudotime in each trajectory, we computed the average gene expression for each GFC. Subsequently, we applied a Savitzky–Golay filter (Dombi and Dineva, 2020) in the Scipy library to smooth the signal curve. The window size was set to 601, which roughly corresponds to 25 intervals along the trajectory, and a polynomial order of three was employed to capture convolution coefficients in the fitted polynomials.

## Result

### The mouse natural killer (mNK) cell differentiation and maturation revealed by cell trajectories with tissue proportion

**FIGURE 2:**
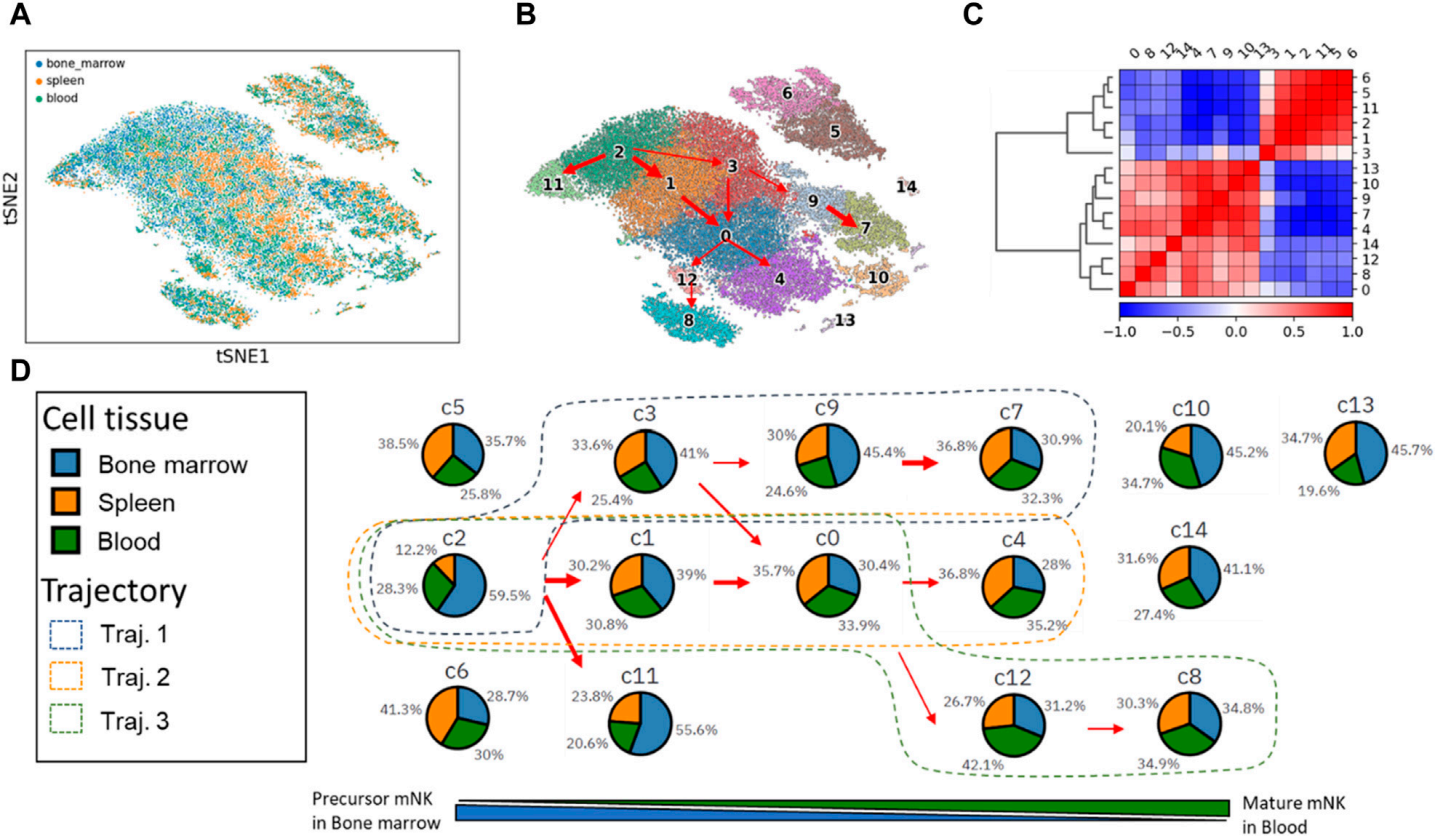
The t-SNE plot of mNK cells from the 3 cell tissues and 15 mNK cell clusters with trajectories inferred from the PAGA map. **(A)** The t-SNE plot of the single cell. **(B)** The 15 cell clusters and trajectories inferred from the PAGA map. **(C)** The correlation matrix of the two super-clusters based on the Leiden cluster’s expression correlation. **(D)** The changes in cell tissue proportion in each of the clusters align with the three trajectories.

We utilized PAGER-scFGA to explore the cell clusters and the trajectories underlying mNK’s maturation and differentiation (Figure 2). Following Leiden clustering and PAGA analysis, we unveiled a total of 15 distinctive clusters. Most notably, a multifurcation pattern became evident, revealing three major trajectories extending from cluster c2 to c7, c4, and c8. These trajectories were elucidated through their interconnected weighted neighborhood relationships.

Notably, we observed the presence of 2 cell subsets based on gene expression correlations within the Leiden cell clusters, mNK precursor cell subset (c2, c3, c1, c11, c5, and c6) and mNK cell mature subset (c9, c0, c4, c7, 12, c8, c10, c14 and c13). Sorting the Leiden clusters based on cell tissue composition revealed these 2 cell subsets, as primarily evident in the correlation matrix. These 2 cell subsets help delineate the underlying cellular relationships and distinctions.

The three major trajectories are visually represented in the Leiden cluster map, with Trajectory one encompassing clusters c2, c3, c9, and c7; Trajectory two involving clusters c2, c1, c0, and c4; and Trajectory three consisting of clusters c2, c1, c0, c12, and c8.

Cluster c2 stands out with the highest proportion of bone marrow tissue (59.5%) and the lowest spleen tissue (12.2%). It is worth noting that natural killer (NK) cells originate from hematopoietic stem cells in the bone marrow. As these NK cells mature in the bone marrow, they subsequently enter the bloodstream as fully functional cells and disseminate throughout various tissues, including the spleen and blood vesicle. Consequently, the prevalence of cluster c2 in precursor mNKs is linked to a high representation of bone marrow. Furthermore, c2 transitions into clusters c7, c4, and c8, marked by a gradual decrease in bone marrow composition and a concurrent increase in spleen and blood tissues.

After the comparison of clusters c7, c4, and c8, we made several key observations regarding the gene expressions in these clusters (as shown in Supplementary Figure S1). Cluster c7 exhibits high expression levels of five genes: Fcer1g, Nkg7, Gzma, Zfp36l2, and Prf1. These genes collectively suggest that c7 represents mature NK cells with the highest cytotoxic activity among the three clusters c7, c4 and c8. Cluster c4 displays moderate expression levels of Ccr2 and Tyrobp in comparison to clusters c8 and c7. Cluster c8 has the lowest expression of the seven genes examined. However, it is distinguished by its high expression of surface glycoproteins (Cd3d, Cd3g, and Cd3e). Additionally, c8 is characterized by high levels of the cell differentiation antigen (Ly6c2) and the receptor for various cytokines (Il7r).

It is worth noting the functions of these genes reported in existing literature. T-cell receptor (TCR)-positive and Fcer1g-expressing innate-like T-cells are known to exhibit high cytotoxic activity (Morrish and Ruland, 2022). Additionally, FCER1G downregulation has been correlated with a loss of immunoregulatory cytotoxic activity in Cd56-CD16^+^ (adaptive) NK cells (Forconi et al., 2020). Nkg7 functions as a regulator of lymphocyte granule exocytosis and downstream inflammation, playing a role in various diseases (Ng et al., 2020). Both Gzma and Prf1 involve cytotoxic lymphocyte-mediated immunity (Zhou et al., 2020) and play critical roles in the NK cells differentiation into efficient killers (Voskoboinik et al., 2015). Zfp36l2 provides a mechanism for attenuating protein synthesis (Adachi et al., 2014; Zheng et al., 2022). Hence, we found that the c7 should be mature NK cells with the highest cytotoxic activity. The c4 shows the mediate expression levels of Ccr2 and Tyrobp compared to the c8 and c7. The c8 has the lowest levels of the seven genes, but shows a high level of surface glycoprotein (Cd3d, Cd3g and Cd3e). The Bcl11b-mediated gain of CD3e, physically associated with CD16 signaling molecules Lck and CD247 in NK cells, is correlated with increased Ab-dependent effector function (Wu et al., 2021). This suggests that cluster c8 may functionally exhibit characteristics reminiscent of a natural killer T-like (NKT) cell.

### IL2 gene expression and IL2 receptor in cell subsets show functional differences between precursor cells and mature cells

**FIGURE 3:**
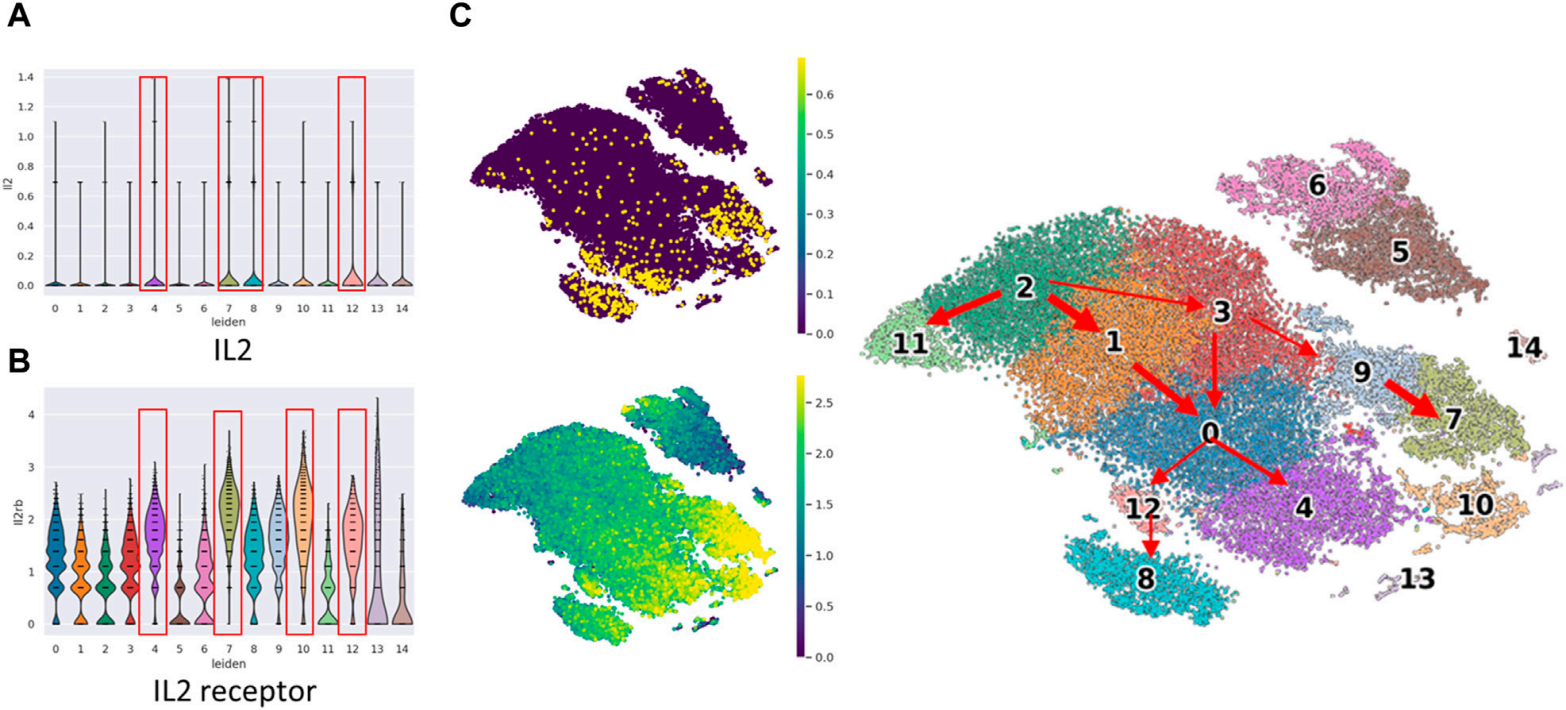
The violin plot of IL2 gene expression and IL2 receptor (IL2RB protein) expression in cell clusters and cell location in t-SNE plot. The violin plot of **(A)** IL2 gene expression and **(B)** IL2RB expression among the 15 cell clusters. The clusters in the red frame are the top four IL2/IL2 receptor expressed clusters, **(C)** The concurrent expression of IL2 gene expression and IL2RB expression in cell clusters.

We conducted an examination of Interleukin-2 (IL2 gene) and IL2RB expression to corroborate the functional activation of NK cells (Figure 3). IL2 is well-known for its capacity to stimulate and activate NK cells, a type of white blood cell responsible for identifying and eliminating infected or cancerous cells. IL2 plays a pivotal role in augmenting the cytotoxic activity of NK cells, thereby enhancing their effectiveness in targeting and eradicating abnormal cells.

Our observations indicated that IL2 gene expression was notably prominent in clusters c4, c7, c8, and c12, which belong to the mature mNK subset. Similarly, IL2RB expression exhibited high levels in clusters c4, c7, c10, and c12, also corresponding to the mature mNK subset. Remarkably, three clusters demonstrated concurrent high expression of both IL2 and IL2 receptor (IL2RB). Notably, these clusters exhibited a relatively low proportion of bone marrow, which did not exceed 32%.

### Functional inference of the cell types among mNK cell clusters show tissue-specific pattern

**FIGURE 4:**
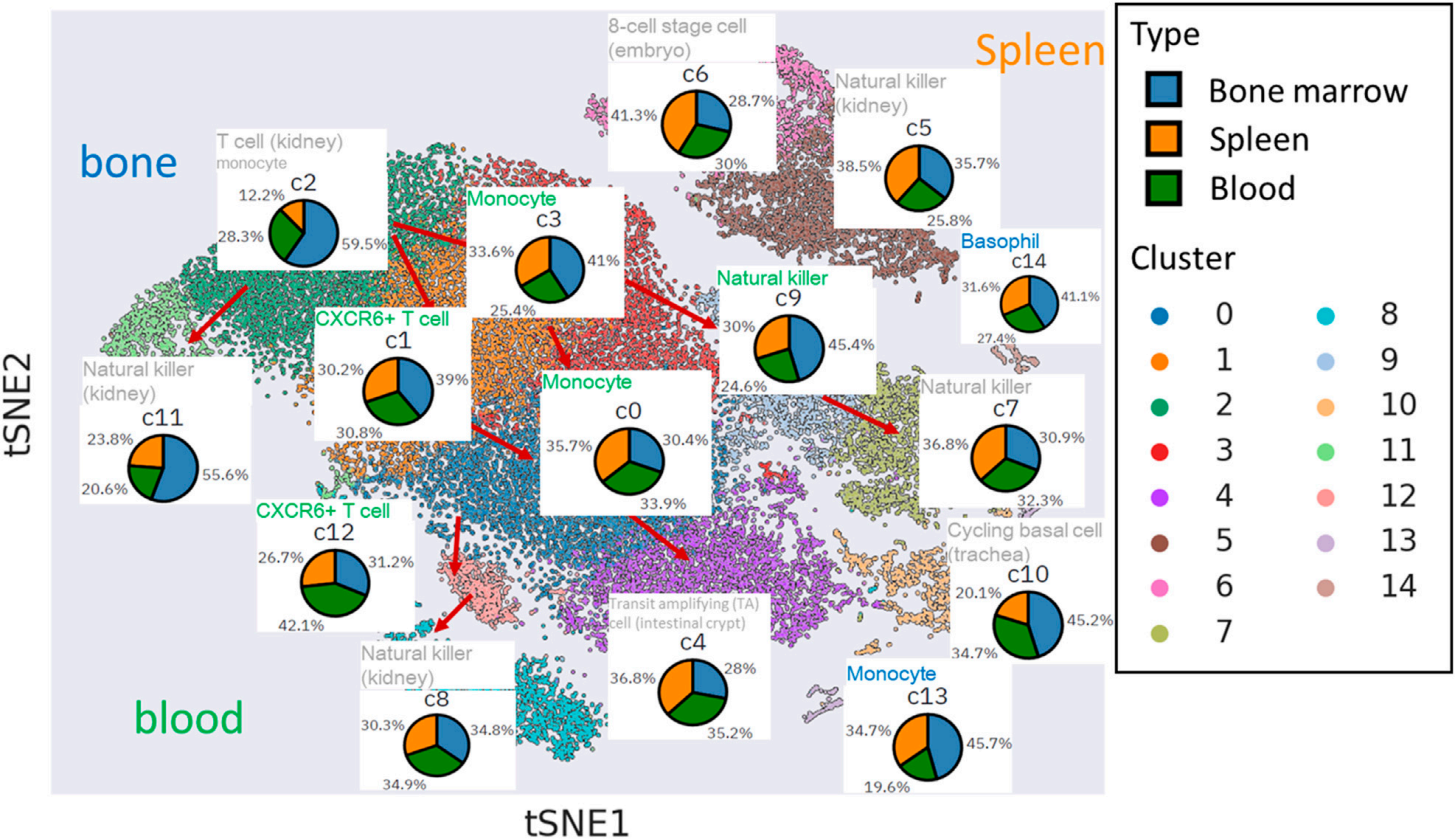
The functional inference of cell type within cell clusters in t-SNE plot. The inferred cell type functions are annotated above the respective cell tissue’s pie chart. The font colors used for annotation vary to signify the tissue of gene signatures enriched by cluster-specific DEGs from mNKs, sourced from the cellmarkers database.

We applied PAGER enrichment analysis using gene signatures sourced from the cellMarkers database (Figure 4). Notably, some of the missed hits, represented in grey, were primarily associated with bone marrow and spleen-related tissues. In contrast, cell clusters linked to blood tissue displayed robust hits through the utilization of differentially expressed genes (DEGs).

Clusters c1, c3, c12, c0, and c9 are intricately connected to blood tissue, particularly in the transition from mNK precursor cells to fully mature mNK cells. For instance, when we examined the topological neighbors of c12 and c1, we found an enrichment of gene signatures associated with CXCR6+ T cells in blood. These cells are characterized by their transition from stem-like states into effector-like cytotoxic T cells (CTLs), involving significant chemotactic reprogramming, including the upregulation of the chemokine receptor CXCR6 as discussed in (Di Pilato et al., 2021). Our discovery of mNK cells suggests that they undergo analogous functional changes to T cells during mNK maturation, thus exhibiting cytotoxic activity akin to NK cells.

Furthermore, our observations revealed that the topological neighbors of c3 and c0 shared functional similarities with monocyte cells in blood. Despite NK cells being a type of lymphocyte derived from a common lymphoid progenitor associated with B and T cells, our findings support the idea that NK cells also exhibit several functional and phenotypic similarities to myeloid progenitor cells, which constitute a significant part of the innate immune response, including monocyte cells (Murin, 2020).

Additionally, our findings revealed that the endpoint clusters (c8, c4, and c7) within the natural killer cell trajectories did not exhibit a distinct tissue-specific enrichment. However, their functionality was primarily associated with the characteristics of mature NK cells commonly found in the kidney (c4 and c7).

### The interplay of identified DEGs in the network underlying the mNK cell maturation and differentiation in the three trajectories

**FIGURE 5:**
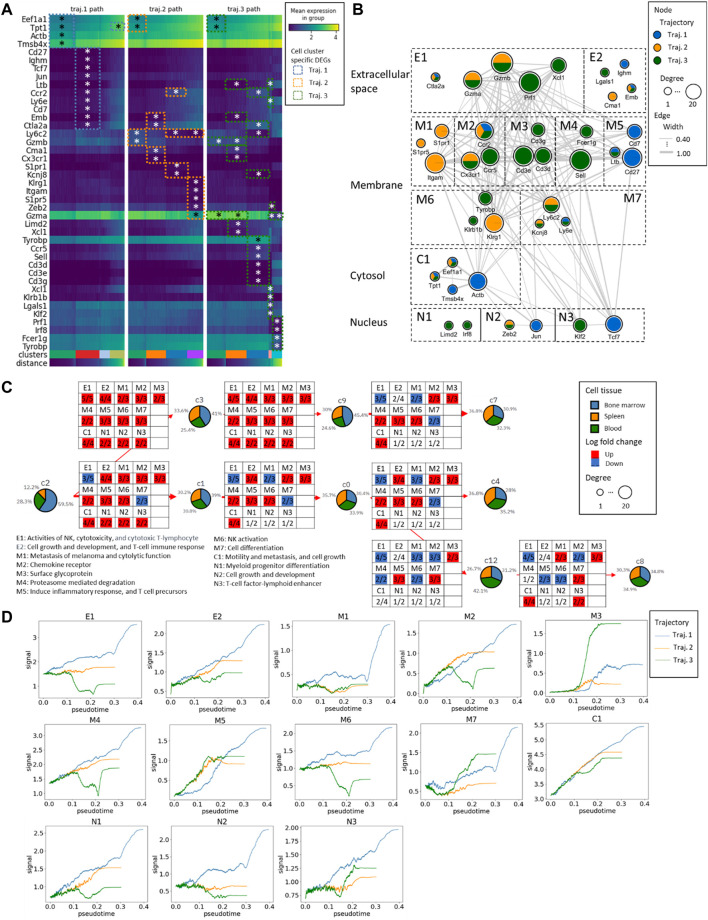
The gene expression alteration aligning with the mNK cell maturation in the three trajectories. **(A)** The heatmap of the expression profiles from DEGs. The * represents the significance of the genes with Wilcoxon *p*-values≤0.05. The distance represents the pseudotime along the PAGA trajectory. **(B)** The Protein-Protein Interaction (PPI) network of DEGs and the gene subcellular map. According to the gene-to-gene connectivity and trajectories, the network has been further split into different functional components, extracellular space components (E1 and E2), membrane components (M1 to M7), cytosol components (C1), and nucleus components (N1 to N3). **(C)** The functional components with an overall number of up/downregulated genes in the NK maturation and differentiation. **(D)** The changes in GFC signal across pseudotime. The blue curve corresponds to trajectory #1, the orange curve to trajectory #2, and the green curve to trajectory #3.

We identified 14 DEGs in trajectory #1, 16 DEGs in trajectory #2, and 30 DEGs in trajectory #3. Notably, there are 5 DEGs (Eef1a1, Tpt1, Ccr2, Emb, and Ctla2a) common to trajectories #1 and #2, 7 DEGs (Eef1a1, Tpt1, Ltb, Ccr2, Ly6e, Emb, and Ctla2a) shared between trajectories #1 and #3, and 5 DEGs (Eef1a1, Tpt1, Ccr2, Emb, and Ctla2a) in common between trajectories #2 and #3 (see Figure 5; Supplementary Figures S2–S4). In the pathway enrichment analysis across the three trajectories, the ribosome pathways stand out as consistently enriched in all of them. Additionally, trajectory #1 exhibits enrichment in cytoskeleton-related and chromatin-related pathways. Trajectory #2, on the other hand, is enriched in G protein-coupled receptor-related pathways, Sphingosine 1-phosphate (S1P) pathways, as well as VEGF, RAS, and PDGFR signaling pathways. Meanwhile, trajectory #3 demonstrates enrichment in T-cell activation pathways, NK cell cytotoxicity pathways, IL signaling, and cytokine pathways (Supplementary Figures S2–S4). This observation is intriguing, suggesting a growing diversity of genes during mNK differentiation, signifying their involvement in activating different molecular mechanisms as cells differentiate.

Further examination included gene grouping based on function and cellular components analysis, which led to the identification of two extracellular components, seven membrane components, one cytosol component, and three nucleus components. In the analysis of DEGs during transitions between different clusters, genes with log fold changes were extracted, and the number of up- and downregulated genes in each gene functional compartment (GFC) was mapped. We found conserved upregulation in the C1 GFC throughout transitions, denoting high activity in cell motility, metastasis, and cell growth, led by genes such as Tpt1, Eef1a1, Actb, and Tmsb4x.

In the bifurcation between trajectory #1 (C2 to C3) and trajectories #2/#3 (C2 to C1), 2 GFCs emerged: E1 (NK activities, cytotoxicity, and cytotoxic T-lymphocytes) and M7 (related to cell differentiation). Similarly, during the transition from C3 to C9, there was an overexpression among all the GFCs. Some compartments, such as M1 (related to melanoma metastasis and cytolytic function), remained blocked in specific transitions, such as C1 to C0, C9 to C7, and C0 to C4. Additionally, during the transition from C0 to C12, two membrane-related compartments, M2 (chemokine receptor) and M4 (proteasome-mediated degradation), were downregulated, along with M6 (NK activation).

Trajectory #1 consistently maintains the highest overall GFC signal levels in psuedotime. In contrast, trajectory #3 exhibits consistently low levels of E1 (related to NK activities, cytotoxicity, and cytotoxic T-lymphocytes), M2 (chemokine receptor), M4 (proteasome-mediated degradation), M6 (NK activation), N1 (myeloid progenitor differentiation), and N2 (cell growth and development). However, it shows a notably elevated level of M3 (surface glycoprotein).

In summary, in the early stages of mNK differentiation, E1 (NK activities, cytotoxicity, and cytotoxic T-lymphocytes) dominated by Prf1, Gzma, and Gzmb, and M7 (related to cell differentiation) dominated by Kcnj8 and Ly6c2 were prominent indicators. In the late stages of mNK differentiation, M2 (chemokine receptor) was dominated by Ccr2, Ccr5, and Cx3cr1, M4 (proteasome-mediated degradation) was dominated by Sell and Fcer1g, and M6 (NK activation) was dominated by Tyrobp, Klrg1, and Klrb1b played significant roles. Cell maturation is characterized by three distinct GFCs, including E1 (related to NK activities, cytotoxicity, and cytotoxic T-lymphocytes) dominated by Prf1, Ctla2a, Gzma, and Gzmb, M1 (related to melanoma metastasis and cytolytic function) dominated by Itgam, S1pr5, and S1pr1, and M7 (related to cell differentiation) dominated by Kcnj8 and Ly6c2. In the comparison of the endpoints of the three trajectories (c7, c4, and c8), it is noteworthy that the number of upregulated GCFs exhibits a positive correlation with the levels of IL2 receptor (Figure 3).

## Discussion and conclusion

The PAGER-scFGA is one of the online interactive single-cell tools tailored to decipher molecular insights into cell heterogeneity using single-cell data. PAGER-scFGA plays a pivotal role in integrating functional genomics analysis into single-cell analysis. The platform offers PAGER enrichment analysis, revealing functional interpretation of DEGs by PAGs. Compared to other tools like ShinyCell (Ouyang et al., 2021), scViewer (Patil et al., 2023) and ICARUS (Jiang et al., 2022), PAGER-scFGA stands out with its advanced analyses, especially in integrating heterogeneous gene sets within PAGER. It enables the extraction of PAG-to-PAG relationships to construct PAG networks and identifies Protein-Protein Interactions (PPIs) among RP-score (Yue et al., 2019; Yue et al., 2021) ranked gene members within retrieved PAGs. Additionally, it facilitates iterative DEG analysis within selected cell clusters, thus enhancing the identification of functional differences along cell trajectories, paving the way for hypothesis-driven research, such as investigating cell differentiation or maturation processes.

We particularly addressed the real-world application in the mouse natural killer (mNK) cell analysis. Within its analytical framework, researchers delve deep into cellular heterogeneity and unearth the underlying DEGs, all while benefiting from cell type inference through enrichment analysis. Furthermore, we showcase the platform’s potential in constructing cell-state-specific networks spanning multiple trajectories, thus enabling the comparative assessment of GFCs (Gene Functional Compartments) and the interpretation of driving molecular mechanisms behind cell maturation and differentiation. As a potential avenue for enhancement, the PAGER-scFGA enrichment analysis could be further empowered by the integration of additional spatial information when the data is available coupled with cell-type inference tools like cellDART (Bae et al., 2022). For the single-cell multimodal study, a promising future direction involves integrating regulatory networks with the availability of ATAC-seq data, and exploration of the cell-state-specific networks.

In the mouse nature killer (mNK) cell analysis, we explored the key GFCs underlying the mNK cell maturation and differentiation. NK cells are innate cytotoxic lymphoid cells (ILCs) involved in the killing of infected and tumor cells. We revealed the two major mNK stages by the cell expression correlation. In the early stage of mNK differentiation, 2 GFCs play a vital role in the divergence of trajectory #1 and trajectories (#2 and #3), including an extracellular GFC dominated by Prf1, Gzma, and Gzmb for NK activities and cytotoxicity, and the GFC dominated by Kcnj8 and Ly6c2 for cell differentiation. In the late stages of mNK differentiation, three membrane GFcs play a vital role in the divergence of trajectory #2 and trajectories #3, including chemokine receptor-related genes (Ccr2, Ccr5, and Cx3cr1), proteasome-mediated degradation-related genes (Sell and Fcer1g), and NK activation related genes (Tyrobp, Klrg1, and Klrb1b).

There are certain limitations present in both PAGER-scFGA and real-world studies. The causal relationships between the GFCs and mNK cell subsets need to be further validated through *in/ex vivo* experiments. Additionally, the PPI networks extracted from either STRING or HAPPI databases are not cell-type-specific. A further implementation comes with gene co-expression pattern mining, gene regulatory relationship inference and cell-type-specific modularity construction. In single-cell analysis of complex diseases, integrating the evaluation of genes annotated as “hot” by GWAS studies could have profound implications. This integration enhances our understanding of the intricate relationship between cells and diseases, as gleaned from databases, such as DisGeNET database (Pinero et al., 2020), GWASdb (Li et al., 2016). We believe that PAGER-scFGA will emerge as a potent tool, shedding light on the signaling events aligned with network analysis. This, in turn, lays a promising foundation for expediting the translational study of complex diseases with the potential to discover reliable cell-state-specific biomarkers and formulate novel hypotheses, such as identifying potential drugs or treatment plans to restore dysregulated cellular states by reverting abnormal expressions to normal levels.

## Data Availability

The original contributions presented in the study are included in the article/Supplementary Material, further inquiries can be directed to the corresponding authors.

## Supplementary material

SUPPLEMENTARY FIGURE S1 Differential expression analysis of the endpoint clusters determines cluster-specific DEGs across c7, c4 and c8. Values represent gene expression. Violin plots of selected DEGs are displayed to the right of the dotplot. The DEGs expressed in each cluster after Wilcoxon’s test and z-score filtering.

SUPPLEMENTARY FIGURE S2 The mNK cell maturation in trajectory #1. **(A)** the cell tissue proportion transition in mNK cell maturation and the underlying DEGs based on a comparison between the selected cluster (e.g., C2) and the combined background clusters (e.g., C2, C3, C9, and C7). **(B)** DEG’s expression overlayed on the cell map and the cell function inferred from WikiGene (https://www.wikigenes.org/). **(C)** Pathway enrichment results and the interconnected pathway cross-talk network are presented, derived from the m-type PAG-PAG relationships facilitated by PAGER. The figure was generated using the Cytoscape software.

SUPPLEMENTARY FIGURE S3 The mNK cell maturation in trajectory #2. **(A)** the cell tissue proportion transition in mNK cell maturation and the underlying DEGs based on a comparison between the selected cluster and the combined background clusters. **(B)** DEG’s expression overlayed on the cell map and the cell function inferred from WikiGene (https://www.wikigenes.org/). **(C)** Pathway enrichment results and the interconnected pathway cross-talk network are presented, derived from the m-type PAG-PAG relationships facilitated by PAGER. The figure was generated using the Cytoscape software.

SUPPLEMENTARY FIGURE S4 The mNK cell maturation in trajectory #3. **(A)** the cell tissue proportion transition in mNK cell maturation and the underlying DEGs based on a comparison between the selected cluster and the combined background clusters. **(B)** DEG’s expression overlayed on the cell map and the cell function inferred from WikiGene (https://www.wikigenes.org/). **(C)** Pathway enrichment results and the interconnected pathway cross-talk network are presented, derived from the m-type PAG-PAG relationships facilitated by PAGER. The figure was generated using the Cytoscape software.

SUPPLEMENTARY TABLE S1 The GO cellular component annotation of the genes in the PPI network.
