# Supplementary material for: PAGER-scFGA: unveiling cell functions and molecular mechanisms in cell trajectories through single-cell functional genomics analysis
Source: Front Bioinform. 2024 Apr 16;4:1336135. doi: 10.3389/fbinf.2024.1336135 (PMC11058213; doi:10.3389/fbinf.2024.1336135)
Supplement: Supplementary file 1 [file DataSheet1.docx]

PAGER-scFGA: Unveiling Cell Functions and Molecular Mechanisms in Cell Trajectories through Single-Cell Functional Genomics Analysis

# Supplementary:

## Filter DEGs in the endpoint clusters of the three trajectories

The selection of DEGs in the endpoint clusters in the three mNK trajectories is based on both the gene's log fold change and its associated z-score underlying the computation of a p-value for each gene for each group within the scanpy.tl.rank_genes_groups function.

Here's a breakdown of the criteria for each endpoint cluster:

We applied an absolute log fold change cutoff of 1.2, and the score cutoff varied from different clusters. We set the c7 specific DEGs score no less than 55, the c4 specific DEGs score no less than 20, and the c8 specific DEGs score to no less than 40.


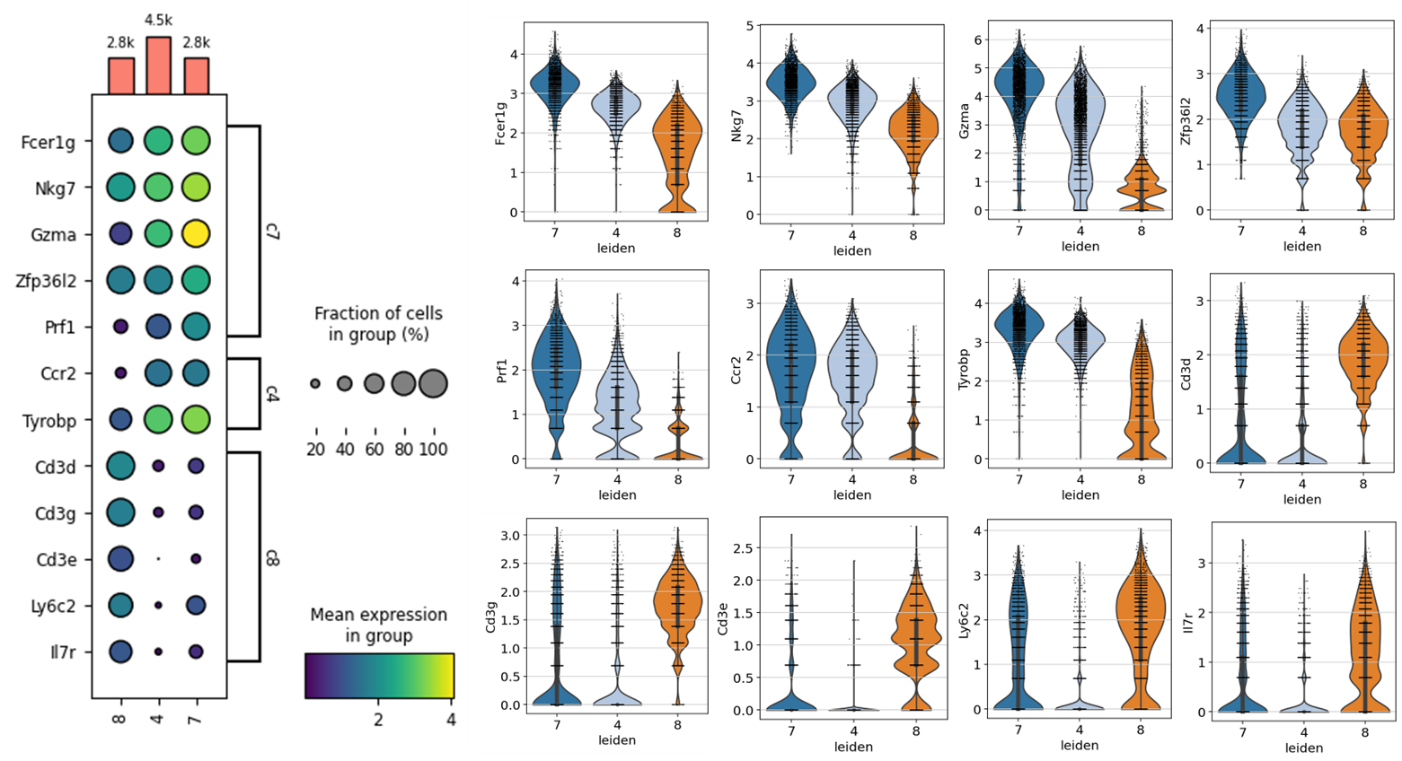


Figure S 1. Differential expression analysis of the endpoint clusters determines cluster-specific DEGs across c7, c4 and c8. Values represent gene expression. Violin plots of selected DEGs are displayed to the right of the dotplot. The DEGs expressed in each cluster after Wilcoxon’s test and z-score filtering.

## Filter DEGs in the selected cluster compared to the reference clusters in the trajectory

The selection of DEGs in each mNK trajectory is based on both the gene's log fold change and its associated z-score, calculated within the scanpy.tl.rank_genes_groups function. Here's a breakdown of the criteria for each trajectory:

In trajectory #1, we applied an absolute log fold change cutoff of 1.1 and the score cutoff varied from different clusters. We set the c2 specific DEGs score either no larger than -78 or no less than 1, the c3 specific DEGs score either no larger than -20 or no less than 1, the c9 specific DEGs score either no larger than -1 or no less than 1, and the c7 specific DEGs score either no larger than -2 or no less than 80.

In trajectory #2, we applied an absolute log fold change cutoff of 1, and the score cutoff varied from different clusters. We set the c2 specific DEGs score either no larger than -88 or no less than 30, the c1 specific DEGs score either no larger than -40 or no less than 6, the c0 specific DEGs score either no larger than -10 or no less than 10, and c4 specific DEGs score either no larger than -20 or no less than 97.

In trajectory #3, we applied an absolute log fold change cutoff of 1 and the score cutoff varied from different clusters. We set the c2 specific DEGs score either no larger than -87 or no less than 30, the c1 specific DEGs score either no larger than -30 or no less than 6, the c0 specific DEGs score either no larger than -15 or no less than 37, the c12 specific DEGs score either no larger than -25 or no less than 34, and the c8 specific DEGs score either no larger than -58 or no less than 80.

**
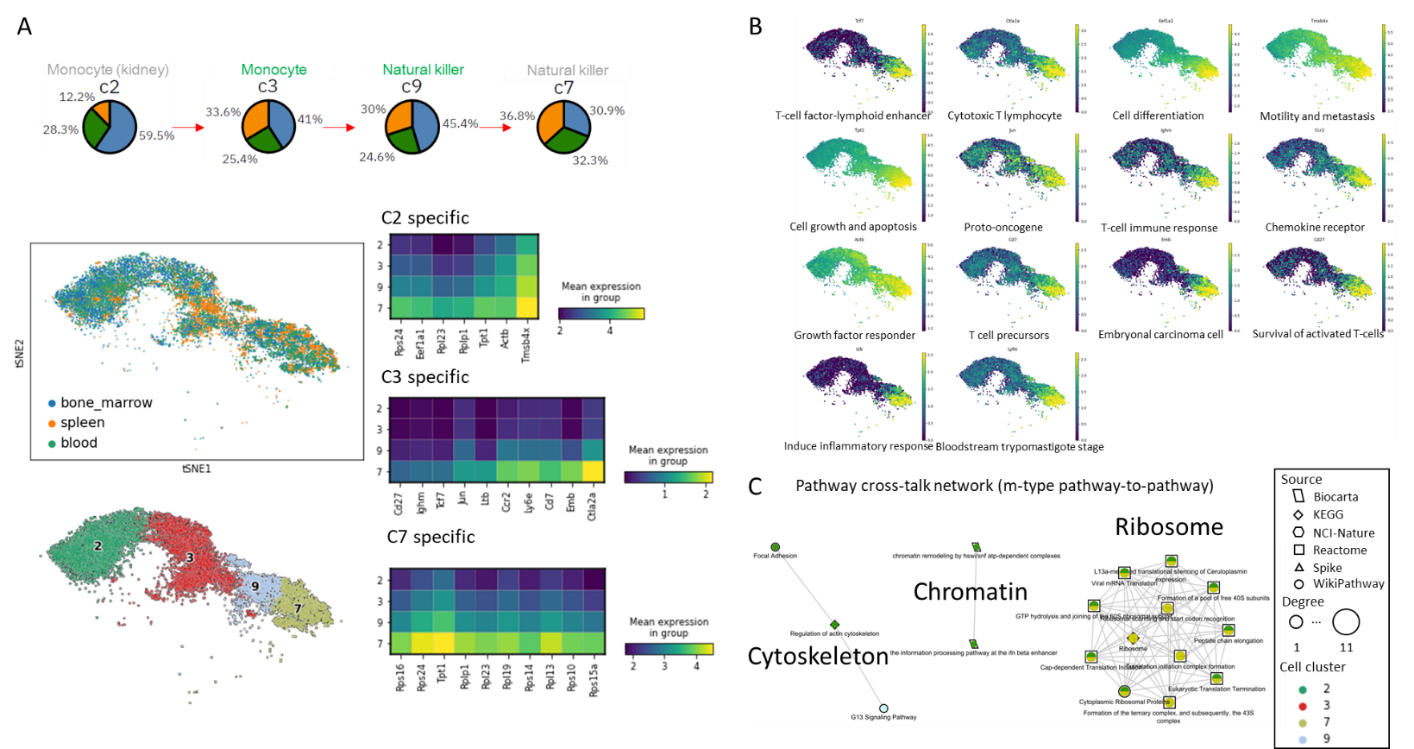
**

Figure S 2. The mNK cell maturation in trajectory #1. (A) the cell tissue proportion transition in mNK cell maturation and the underlying DEGs based on a comparison between the selected cluster (e.g., C2) and the combined background clusters (e.g., C2, C3, C9, and C7). (B) DEG’s expression overlayed on the cell map and the cell function inferred from WikiGene (https://www.wikigenes.org/). (C) Pathway enrichment results and the interconnected pathway cross-talk network are presented, derived from the m-type PAG-PAG relationships facilitated by PAGER. The figure was generated using the Cytoscape software.

**
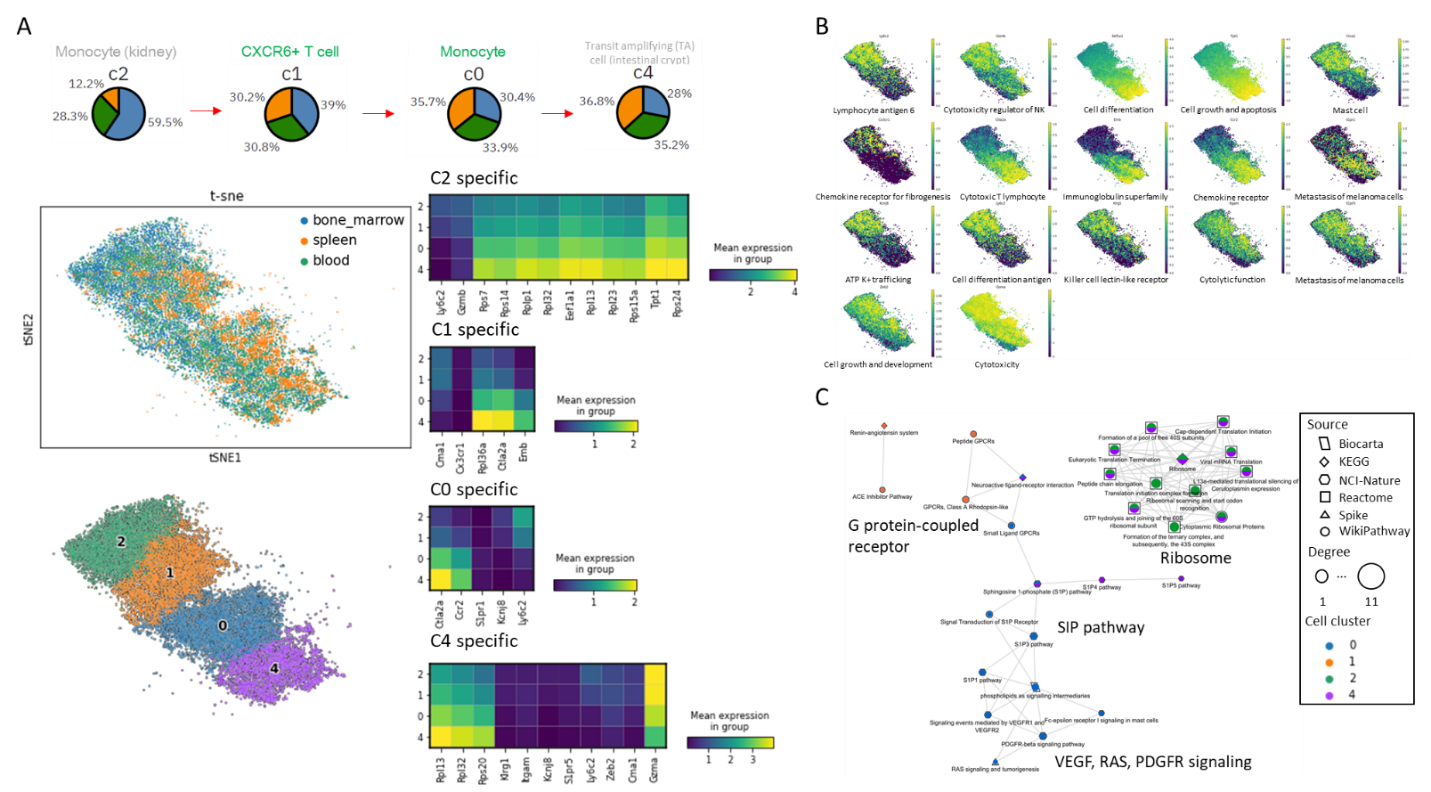
**

Figure S 3. The mNK cell maturation in trajectory #2. (A) the cell tissue proportion transition in mNK cell maturation and the underlying DEGs based on a comparison between the selected cluster and the combined background clusters. (B) DEG’s expression overlayed on the cell map and the cell function inferred from WikiGene (https://www.wikigenes.org/). (C) Pathway enrichment results and the interconnected pathway cross-talk network are presented, derived from the m-type PAG-PAG relationships facilitated by PAGER. The figure was generated using the Cytoscape software.

**
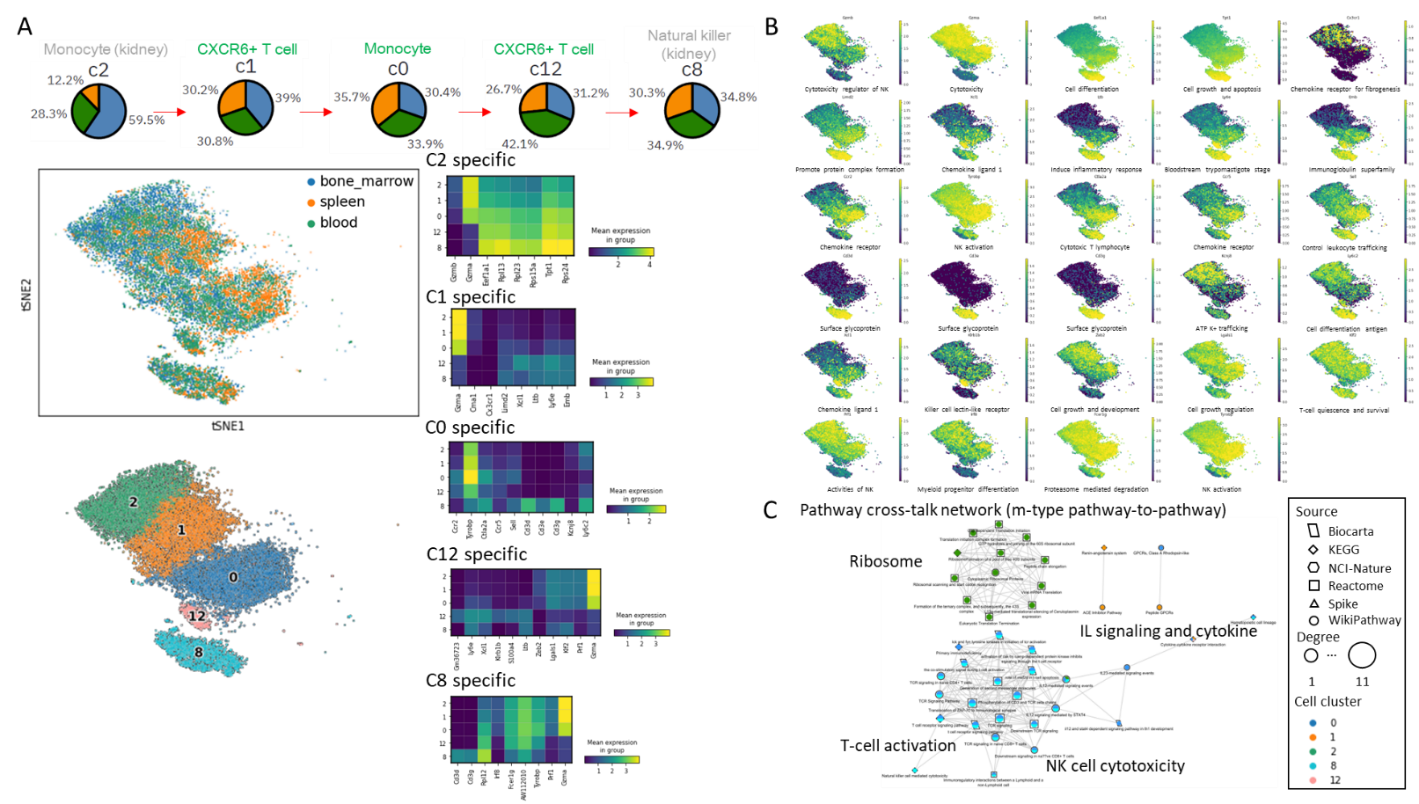
**

Figure S 4. The mNK cell maturation in trajectory #3. (A) the cell tissue proportion transition in mNK cell maturation and the underlying DEGs based on a comparison between the selected cluster and the combined background clusters. (B) DEG’s expression overlayed on the cell map and the cell function inferred from WikiGene (https://www.wikigenes.org/). (C) Pathway enrichment results and the interconnected pathway cross-talk network are presented, derived from the m-type PAG-PAG relationships facilitated by PAGER. The figure was generated using the Cytoscape software.

Table S 1 The GO cellular component annotation of the genes in the PPI network.

| **Symbol** | **GOID** | **name** | **type** | **description** | **link** |
| --- | --- | --- | --- | --- | --- |
| **Actb** | **GO:0015629** | **actin cytoskeleton** | **cellular_component** | **The part of the cytoskeleton (the internal framework of a cell) composed of actin and associated proteins. Includes actin cytoskeleton-associated complexes.** | **http://amigo.geneontology.org/amigo/search/ontology?q=GO:0015629** |
| **Ccr2** | **GO:0009897** | **external side of plasma membrane** | **cellular_component** | **The leaflet of the plasma membrane that faces away from the cytoplasm and any proteins embedded or anchored in it or attached to its surface.** | **http://amigo.geneontology.org/amigo/search/ontology?q=GO:0009897** |
| **Ccr5** | **GO:0009897** | **external side of plasma membrane** | **cellular_component** | **The leaflet of the plasma membrane that faces away from the cytoplasm and any proteins embedded or anchored in it or attached to its surface.** | **http://amigo.geneontology.org/amigo/search/ontology?q=GO:0009897** |
| **Cd27** | **GO:0009897** | **external side of plasma membrane** | **cellular_component** | **The leaflet of the plasma membrane that faces away from the cytoplasm and any proteins embedded or anchored in it or attached to its surface.** | **http://amigo.geneontology.org/amigo/search/ontology?q=GO:0009897** |
| **Cd3d** | **GO:0009897** | **external side of plasma membrane** | **cellular_component** | **The leaflet of the plasma membrane that faces away from the cytoplasm and any proteins embedded or anchored in it or attached to its surface.** | **http://amigo.geneontology.org/amigo/search/ontology?q=GO:0009897** |
| **Cd3e** | **GO:0042101** | **T cell receptor complex** | **cellular_component** | **A protein complex that contains a disulfide-linked heterodimer of T cell receptor (TCR) chains, which are members of the immunoglobulin superfamily, and mediates antigen recognition, ultimately resulting in T cell activation. The TCR heterodimer is associated with the CD3 complex, which consists of the nonpolymorphic polypeptides gamma, delta, epsilon, zeta, and, in some cases, eta (an RNA splice variant of zeta) or Fc epsilon chains.** | **http://amigo.geneontology.org/amigo/search/ontology?q=GO:0042101** |
| **Cd3g** | **GO:0009897** | **external side of plasma membrane** | **cellular_component** | **The leaflet of the plasma membrane that faces away from the cytoplasm and any proteins embedded or anchored in it or attached to its surface.** | **http://amigo.geneontology.org/amigo/search/ontology?q=GO:0009897** |
| **Cd7** | **GO:0016021** | **integral component of membrane** | **cellular_component** | **The component of a membrane consisting of the gene products and protein complexes having at least some part of their peptide sequence embedded in the hydrophobic region of the membrane.** | **http://amigo.geneontology.org/amigo/search/ontology?q=GO:0016021** |
| **Cma1** | **GO:0005615** | **extracellular space** | **cellular_component** | **That part of a multicellular organism outside the cells proper, usually taken to be outside the plasma membranes, and occupied by fluid.** | **http://amigo.geneontology.org/amigo/search/ontology?q=GO:0005615** |
| **Ctla2a** | **GO:0005576** | **extracellular region** | **cellular_component** | **The space external to the outermost structure of a cell. For cells without external protective or external encapsulating structures this refers to space outside of the plasma membrane. This term covers the host cell environment outside an intracellular parasite.** | **http://amigo.geneontology.org/amigo/search/ontology?q=GO:0005576** |
| **Cx3cr1** | **GO:0009897** | **external side of plasma membrane** | **cellular_component** | **The leaflet of the plasma membrane that faces away from the cytoplasm and any proteins embedded or anchored in it or attached to its surface.** | **http://amigo.geneontology.org/amigo/search/ontology?q=GO:0009897** |
| **Eef1a1** | **GO:0030864** | **cortical actin cytoskeleton** | **cellular_component** | **The portion of the actin cytoskeleton, comprising filamentous actin and associated proteins, that lies just beneath the plasma membrane.** | **http://amigo.geneontology.org/amigo/search/ontology?q=GO:0030864** |
| **Emb** | **GO:0005887** | **integral component of plasma membrane** | **cellular_component** | **The component of the plasma membrane consisting of the gene products and protein complexes having at least some part of their peptide sequence embedded in the hydrophobic region of the membrane.** | **http://amigo.geneontology.org/amigo/search/ontology?q=GO:0005887** |
| **Fcer1g** | **GO:0009897** | **external side of plasma membrane** | **cellular_component** | **The leaflet of the plasma membrane that faces away from the cytoplasm and any proteins embedded or anchored in it or attached to its surface.** | **http://amigo.geneontology.org/amigo/search/ontology?q=GO:0009897** |
| **Gzma** | **GO:0005576** | **extracellular region** | **cellular_component** | **The space external to the outermost structure of a cell. For cells without external protective or external encapsulating structures this refers to space outside of the plasma membrane. This term covers the host cell environment outside an intracellular parasite.** | **http://amigo.geneontology.org/amigo/search/ontology?q=GO:0005576** |
| **Gzmb** | **GO:0005576** | **extracellular region** | **cellular_component** | **The space external to the outermost structure of a cell. For cells without external protective or external encapsulating structures this refers to space outside of the plasma membrane. This term covers the host cell environment outside an intracellular parasite.** | **http://amigo.geneontology.org/amigo/search/ontology?q=GO:0005576** |
| **Ighm** | **GO:0005615** | **extracellular space** | **cellular_component** | **That part of a multicellular organism outside the cells proper, usually taken to be outside the plasma membranes, and occupied by fluid.** | **http://amigo.geneontology.org/amigo/search/ontology?q=GO:0005615** |
| **Irf8** | **GO:0005634** | **nucleus** | **cellular_component** | **A membrane-bounded organelle of eukaryotic cells in which chromosomes are housed and replicated. In most cells, the nucleus contains all of the cell's chromosomes except the organellar chromosomes, and is the site of RNA synthesis and processing. In some species, or in specialized cell types, RNA metabolism or DNA replication may be absent.** | **http://amigo.geneontology.org/amigo/search/ontology?q=GO:0005634** |
| **Itgam** | **GO:0009897** | **external side of plasma membrane** | **cellular_component** | **The leaflet of the plasma membrane that faces away from the cytoplasm and any proteins embedded or anchored in it or attached to its surface.** | **http://amigo.geneontology.org/amigo/search/ontology?q=GO:0009897** |
| **Jun** | **GO:0005654** | **nucleoplasm** | **cellular_component** | **That part of the nuclear content other than the chromosomes or the nucleolus.** | **http://amigo.geneontology.org/amigo/search/ontology?q=GO:0005654** |
| **Kcnj8** | **GO:0016021** | **integral component of membrane** | **cellular_component** | **The component of a membrane consisting of the gene products and protein complexes having at least some part of their peptide sequence embedded in the hydrophobic region of the membrane.** | **http://amigo.geneontology.org/amigo/search/ontology?q=GO:0016021** |
| **Klf2** | **GO:0005634** | **nucleus** | **cellular_component** | **A membrane-bounded organelle of eukaryotic cells in which chromosomes are housed and replicated. In most cells, the nucleus contains all of the cell's chromosomes except the organellar chromosomes, and is the site of RNA synthesis and processing. In some species, or in specialized cell types, RNA metabolism or DNA replication may be absent.** | **http://amigo.geneontology.org/amigo/search/ontology?q=GO:0005634** |
| **Klrb1b** | **GO:0016021** | **integral component of membrane** | **cellular_component** | **The component of a membrane consisting of the gene products and protein complexes having at least some part of their peptide sequence embedded in the hydrophobic region of the membrane.** | **http://amigo.geneontology.org/amigo/search/ontology?q=GO:0016021** |
| **Klrg1** | **GO:0043231** | **intracellular membrane-bounded organelle** | **cellular_component** | **Organized structure of distinctive morphology and function, bounded by a single or double lipid bilayer membrane and occurring within the cell. Includes the nucleus, mitochondria, plastids, vacuoles, and vesicles. Excludes the plasma membrane.** | **http://amigo.geneontology.org/amigo/search/ontology?q=GO:0043231** |
| **Lgals1** | **GO:0005615** | **extracellular space** | **cellular_component** | **That part of a multicellular organism outside the cells proper, usually taken to be outside the plasma membranes, and occupied by fluid.** | **http://amigo.geneontology.org/amigo/search/ontology?q=GO:0005615** |
| **Limd2** | **GO:0005634** | **nucleus** | **cellular_component** | **A membrane-bounded organelle of eukaryotic cells in which chromosomes are housed and replicated. In most cells, the nucleus contains all of the cell's chromosomes except the organellar chromosomes, and is the site of RNA synthesis and processing. In some species, or in specialized cell types, RNA metabolism or DNA replication may be absent.** | **http://amigo.geneontology.org/amigo/search/ontology?q=GO:0005634** |
| **Ltb** | **GO:0005615** | **extracellular space** | **cellular_component** | **That part of a multicellular organism outside the cells proper, usually taken to be outside the plasma membranes, and occupied by fluid.** | **http://amigo.geneontology.org/amigo/search/ontology?q=GO:0005615** |
| **Ly6c2** | **GO:0031225** | **anchored component of membrane** | **cellular_component** | **The component of a membrane consisting of the gene products that are tethered to the membrane only by a covalently attached anchor, such as a lipid group that is embedded in the membrane. Gene products with peptide sequences that are embedded in the membrane are excluded from this grouping.** | **http://amigo.geneontology.org/amigo/search/ontology?q=GO:0031225** |
| **Ly6e** | **GO:0031225** | **anchored component of membrane** | **cellular_component** | **The component of a membrane consisting of the gene products that are tethered to the membrane only by a covalently attached anchor, such as a lipid group that is embedded in the membrane. Gene products with peptide sequences that are embedded in the membrane are excluded from this grouping.** | **http://amigo.geneontology.org/amigo/search/ontology?q=GO:0031225** |
| **Prf1** | **GO:0005615** | **extracellular space** | **cellular_component** | **That part of a multicellular organism outside the cells proper, usually taken to be outside the plasma membranes, and occupied by fluid.** | **http://amigo.geneontology.org/amigo/search/ontology?q=GO:0005615** |
| **S1pr1** | **GO:0031226** | **intrinsic component of plasma membrane** | **cellular_component** | **The component of the plasma membrane consisting of the gene products and protein complexes having either part of their peptide sequence embedded in the hydrophobic region of the membrane or some other covalently attached group such as a GPI anchor that is similarly embedded in the membrane.** | **http://amigo.geneontology.org/amigo/search/ontology?q=GO:0031226** |
| **S1pr5** | **GO:0016021** | **integral component of membrane** | **cellular_component** | **The component of a membrane consisting of the gene products and protein complexes having at least some part of their peptide sequence embedded in the hydrophobic region of the membrane.** | **http://amigo.geneontology.org/amigo/search/ontology?q=GO:0016021** |
| **Sell** | **GO:0009897** | **external side of plasma membrane** | **cellular_component** | **The leaflet of the plasma membrane that faces away from the cytoplasm and any proteins embedded or anchored in it or attached to its surface.** | **http://amigo.geneontology.org/amigo/search/ontology?q=GO:0009897** |
| **Tcf7** | **GO:0005634** | **nucleus** | **cellular_component** | **A membrane-bounded organelle of eukaryotic cells in which chromosomes are housed and replicated. In most cells, the nucleus contains all of the cell's chromosomes except the organellar chromosomes, and is the site of RNA synthesis and processing. In some species, or in specialized cell types, RNA metabolism or DNA replication may be absent.** | **http://amigo.geneontology.org/amigo/search/ontology?q=GO:0005634** |
| **Tmsb4x** | **GO:0005856** | **cytoskeleton** | **cellular_component** | **Any of the various filamentous elements that form the internal framework of cells, and typically remain after treatment of the cells with mild detergent to remove membrane constituents and soluble components of the cytoplasm. The term embraces intermediate filaments, microfilaments, microtubules, the microtrabecular lattice, and other structures characterized by a polymeric filamentous nature and long-range order within the cell. The various elements of the cytoskeleton not only serve in the maintenance of cellular shape but also have roles in other cellular functions, including cellular movement, cell division, endocytosis, and movement of organelles.** | **http://amigo.geneontology.org/amigo/search/ontology?q=GO:0005856** |
| **Tpt1** | **GO:0005881** | **cytoplasmic microtubule** | **cellular_component** | **Any microtubule in the cytoplasm of a cell.** | **http://amigo.geneontology.org/amigo/search/ontology?q=GO:0005881** |
| **Tyrobp** | **GO:0016021** | **integral component of membrane** | **cellular_component** | **The component of a membrane consisting of the gene products and protein complexes having at least some part of their peptide sequence embedded in the hydrophobic region of the membrane.** | **http://amigo.geneontology.org/amigo/search/ontology?q=GO:0016021** |
| **Xcl1** | **GO:0005615** | **extracellular space** | **cellular_component** | **That part of a multicellular organism outside the cells proper, usually taken to be outside the plasma membranes, and occupied by fluid.** | **http://amigo.geneontology.org/amigo/search/ontology?q=GO:0005615** |
| **Zeb2** | **GO:0005634** | **nucleus** | **cellular_component** | **A membrane-bounded organelle of eukaryotic cells in which chromosomes are housed and replicated. In most cells, the nucleus contains all of the cell's chromosomes except the organellar chromosomes, and is the site of RNA synthesis and processing. In some species, or in specialized cell types, RNA metabolism or DNA replication may be absent.** | **http://amigo.geneontology.org/amigo/search/ontology?q=GO:0005634** |
